# Predictive value of the Hemoglobin, Albumin, Lymphocyte and Platelet score for mortality in geriatric patients presenting to the emergency department

**DOI:** 10.1111/ggi.15082

**Published:** 2025-01-21

**Authors:** Fatma Tortum, Erdal Tekin, Ali Gur

**Affiliations:** ^1^ Department of Emergency Medicine, School of Medicine Ataturk University Erzurum Turkey

**Keywords:** emergency department, geriatric patients, HALP score, mortality, scoring systems

## Abstract

**Aim:**

The Hemoglobin, Albumin, Lymphocyte and Platelet (HALP) score, calculated as hemoglobin × albumin × lymphocytes / platelets, serves as a novel biomarker that can provide insights into a patient's nutritional status, anemia status and inflammatory processes. This study aimed to investigate the predictive value of the HALP score for mortality among geriatric patients presenting to the emergency department.

**Methods:**

This retrospective study was carried out at the emergency department of a tertiary hospital. Patients aged ≥65 years who presented to the emergency department between 1 January 2018 and 1 January 2024 were included in the study. A total of 62 262 patients who visited our emergency department were enrolled. Patient data, including hemoglobin, albumin, lymphocyte and platelet values; age; sex, the reason for hospital presentation; and outcome (mortality or discharge) were obtained from electronic medical records. HALP scores were calculated for the patients, and statistical analyses were carried out.

**Results:**

Of the patients, 32 410 were men, and the mean age was 73 years. Within this cohort, in‐hospital mortality occurred in 3093 of the patients. The HALP score was significantly lower in patients who died compared with those who were discharged (*P* < 0.001).

**Conclusion:**

Due to its cost‐effectiveness and ease of calculation, the HALP score appears to be more feasible in predicting mortality in the emergency department compared with other scoring systems. **Geriatr Gerontol Int 2025; 25: 387–391**.

## Graphical abstract

A total of 125 965 patients were initially screened. Of these, 25 456 patients due to trauma‐related, 896 patients due to COVID‐19, 2300 patients due to Crimean–Congo hemorrhagic fever, 21 184 patients due to malignancy and 10 971 patients due to incomplete data were excluded. As a result, 62 262 patients were included.

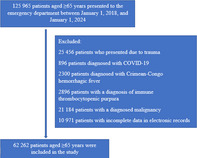

## Introduction

As life expectancy continues to increase, emergency departments are encountering a growing population of geriatric patients. This demographic shift necessitates adjustments in emergency services, and even hospital and health policies in a broader sense.^1^ Predicting mortality among geriatric patients presenting to the emergency department constitutes a complex issue. These patients often face an elevated risk of mortality due to multiple chronic illnesses, polypharmacy and reduced physical activity. To predict mortality, scoring systems, such as Acute Physiology and Chronic Health Evaluation III (APACHE III), Hospice in End‐Stage Liver Disease Prognostic score (HELP scale), Burden of Illness Score for Elderly Persons (BISEP score), Frail Elderly Subject: Evaluation and Follow up (Sujet Âgé Fragile: Évaluation et Suivi‐ SAFES) and Hospital‐Patient One‐Year Mortality Risk (HOMR score), have been developed. Nevertheless, the applicability of these factors to unselected geriatric patients requiring acute critical care is complicated due to variations in performance across population groups and their reliance on the patient's prior medical history, which is often difficult to access and of uncertain reliability in emergency care settings.^2^ Therefore, there is a need for a scale that incorporates more personalized factors; for example, nutritional, as well as acute conditions, such as anemia, to predict mortality among geriatric patients presenting to the emergency department.

The Hemoglobin, Albumin, Lymphocyte and Platelet (HALP) score has garnered attention recently as a scoring system that provides information about patients' nutritional status, anemia status, and inflammatory processes.^3^ Introduced by Chen *et al*. in 2015, the HALP score is calculated as hemoglobin × albumin × lymphocytes / platelets,^4^ and has been frequently utilized in determining prognosis, particularly among patients with malignancies.^3^, ^4^, ^5^, ^6^ In addition, the HALP score has been shown to be valuable for predicting hospital mortality among patients with ST‐segment elevation myocardial infarction, and projecting the occurrence of recurrent strokes and mortality in those with acute ischemic stroke.^7^, ^8^

Considering that the HALP score consists of albumin levels reflecting nutritional and inflammatory status, hemoglobin levels showing anemia status, and lymphocyte and platelet levels showing inflammatory status, it seems to be appropriate for use in the emergency department for geriatric patients. The present study aimed to investigate the predictive value of the HALP score for mortality among geriatric patients presenting to the emergency department.

## Methods

### Study design and data collection

The present retrospective study was carried out in the emergency department of a tertiary hospital. This study was approved by the Ataturk University Clinical Research Ethics Committee with decision number 4/34 and dated 6 July 2024. Patients aged ≥65 years who presented to the emergency department between 1 January 2018 and 1 January 2024 were included in the study. Patients with known malignancies (including hematological malignancies), those who had received chemotherapy or radiotherapy within the past year due to malignancy, those with diagnoses or suspected diagnoses of COVID‐19, those with Crimean–Congo hemorrhagic fever or immune thrombocytopenic purpura, those presenting due to trauma regardless of cause (e.g. falls, motor vehicle accidents, neglect, abuse and crush injuries) and those with incomplete data for any reason were excluded from the study. Data on patient age, sex, date of presentation to the emergency department, provisional diagnosis at presentation, emergency department outcome (hospitalization, discharge or death in the emergency department), diagnosis at the time of admission for hospitalized patients and clinical outcomes (discharge or mortality) were obtained from electronic medical records. Hemogram parameters, including hemoglobin, albumin, lymphocytes and platelets, measured at the time of emergency department presentation were also retrieved from these records.

**Figure 1:**
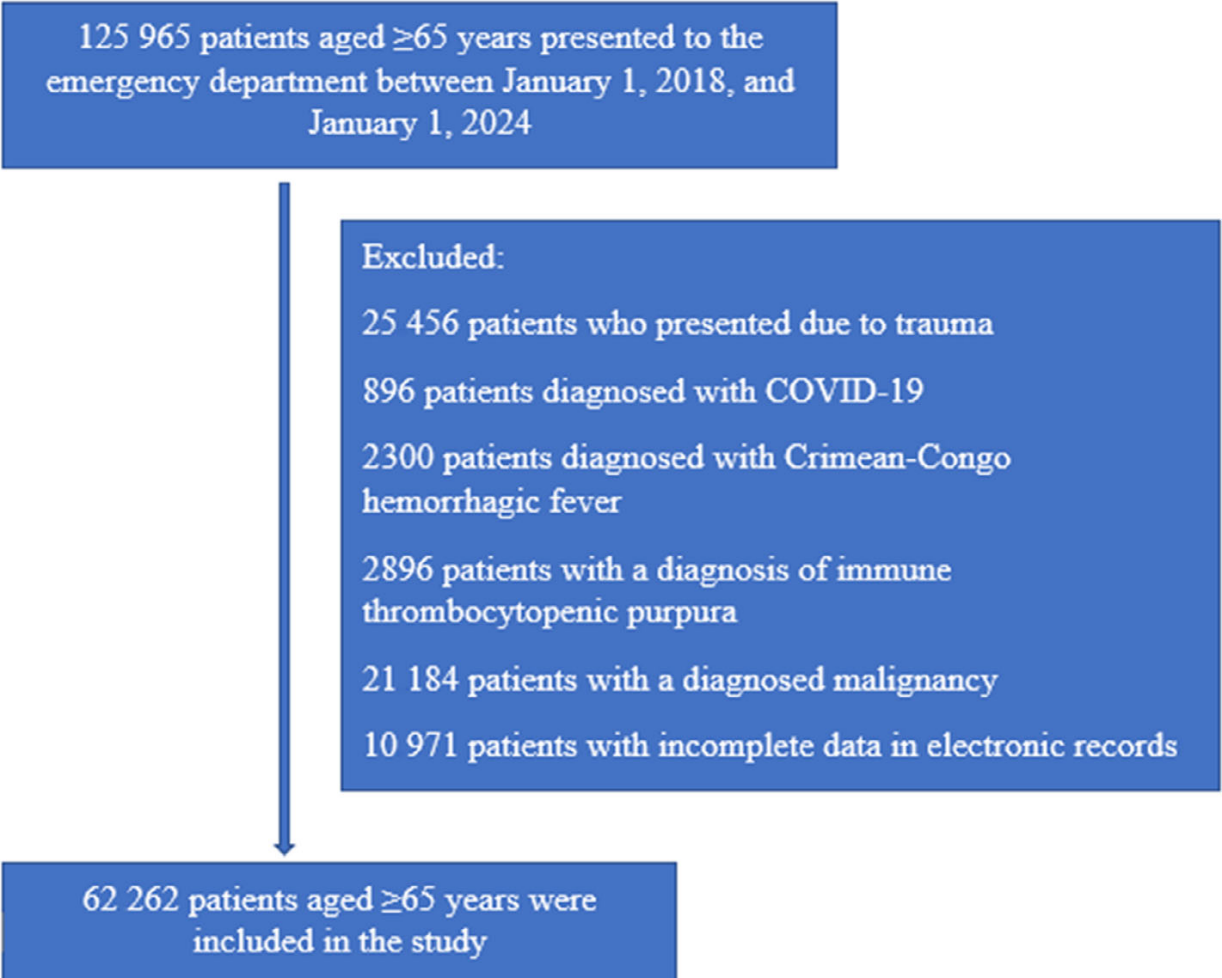
Flow chart for patient selection.

A total of 125 965 patients presenting to the emergency department of Ataturk University Research Hospital Emergency Medicine, Erzurum/Turkey were initially screened. Among these, 25 456 patients presented due to trauma‐related causes (e.g. falls, motor vehicle accidents, neglect, abuse or crush injuries) and were therefore excluded from the study. Further excluded from the study were 896 patients diagnosed with COVID‐19, who were originally admitted for reasons other than COVID‐19 during the pandemic, but received a COVID‐19 diagnosis after hospitalization; 2300 patients diagnosed with Crimean–Congo hemorrhagic fever; and 21 184 patients diagnosed with malignancy. Finally, 10 971 patients were excluded due to incomplete data in their electronic medical records. As a result, 62 262 patients who met the inclusion criteria were included in the sample (Fig. 1).

The data derived from electronic records included hemoglobin (g/dL), albumin (g/dL), lymphocyte count (×10^3^/μL) and platelet count (×10^3^/μL). These parameters were converted to appropriate units (g/L or ×10^3^/μL) for the calculation of the HALP score using the following formula: hemoglobin × albumin × lymphocyte count / platelet count.^4^

### Statistical analysis

Statistical analyses were carried out using SPSS version 25 (IBM, Armonk, NY, USA). The Kolmogorov–Smirnov test was carried out to assess the normality of the data distribution. Descriptive statistics are presented as frequencies (*n*) and percentages (%) for categorical variables, and as median and interquartile range (25%–75%) values for variables without a normal distribution. Comparisons between χ^2^‐test and Fisher's exact test where appropriate. Group comparisons for non‐normally distributed variables were analyzed using the Mann–Whitney *U*‐test. Spearman correlation analysis was used to investigate relationships between variables that did not show a normal distribution.

Receiver operating characteristic analysis was carried out to evaluate the predictive power of the HALP score for in‐hospital mortality and discharge. The area under the receiver operating characteristic curve was calculated for albumin, lymphocyte count and the HALP score for the prediction of patient outcome. Youden's J index was used to determine optimal cut‐off values. Sensitivity and specificity were computed with 95% confidence intervals (CIs). Statistical significance was set at *P* < 0.05.

## Results

The study included a total of 62 262 patients, comprising 32 410 men and 29 852 women. The mean age of the patients enrolled was 73 years. Among these patients, 3093 died in hospital, with a mean age of 77 years. The mean age of patients who experienced mortality was statistically significantly higher compared with those who were discharged (*P* < 0.001). Furthermore, patients in the mortality group had significantly lower HALP scores compared with those who were discharged (*P* < 0.001). Table 1 summarizes the demographic characteristics of the patients, including age, sex, platelet count, hemoglobin level, albumin level, lymphocyte count and HALP scores, categorized by outcome (mortality or discharge).

**Table 1:** Basic characteristics of patients by outcome

Variables	Total (*n* = 62 262)	Mortality (*n* = 3093)	Discharge (*n* = 59 169)	*P*‐value
Median age, years (IQR)	73 (69–79)	77 (71–83)	73 (69–79)	**<0.001**
Sex, *n* (%)				**0.769**
Male	32 410 (52.1%)	1618 (52.3%)	30 792 (52.0%)	
Female	29 852 (47.9%)	1475 (47.7%)	28 377 (48.0%)	
Median platelet count, ×10^3^ μL (IQR)	236 (191–288)	230 (173–300)	236 (192–288)	**<0.001**
Median hemoglobin, g/dL (IQR)	13.3 (11.8–14.7)	12.2 (10.2–14.1)	13.2 (11.8–14.7)	**<0.001**
Median albumin, g/dL (IQR)	3.75 (3.42–4.03)	3.2 (2.7–3.6)	3.8 (3.4–4.0)	**<0.001**
Median lymphocyte count, ×10^3^ μL (IQR)	1.78 (1.02–2.09)	1.1 (0.7–1.8)	1.5 (1.0–2.1)	**<0.001**
Median HALP score (IQR)	37 (29–43)	17.0 (9.2–31.7)	39.2 (18.8–47.5)	**<0.001**

*Note*: Bold indicates significant values (*P* < 0.005).

HALP, Hemoglobin, Albumin, Lymphocyte and Platelet; IQR, interquartile range.

When evaluating the correlation between patient outcomes (mortality or discharge) and other variables, a negative correlation was observed between the HALP score and patient outcome. There was also a negative correlation between age and patient outcome. Both age and the HALP score showed statistically significant relationships with patient outcome (*P* < 0.001 for both). The correlations of the patient outcome with age, sex, platelet count, lymphocyte count, albumin level, hemoglobin level and the HALP score assessed at the time of emergency department presentation are summarized in Table 2.

**Table 2:** Correlations between patient outcome and other variables

Variables		Age	Sex	Plt (×10^3^ μL)	Hb (g/dL)	Albumin (g/dL)	Lymph (×10^3^μL)	HALP score
Patient outcome	r	−0.095	0.001	0.000	0.095	0.235	0.005	0.016
	*P*	<0.001	0.769	0.964	<0.001	<0.001	0.187	<0.001

HALP, Hemoglobin, Albumin, Lymphocyte and Platelet; Hb, hemoglobin; Lymph, lymphocyte count; Plt, platelet count.

**Figure 2:**
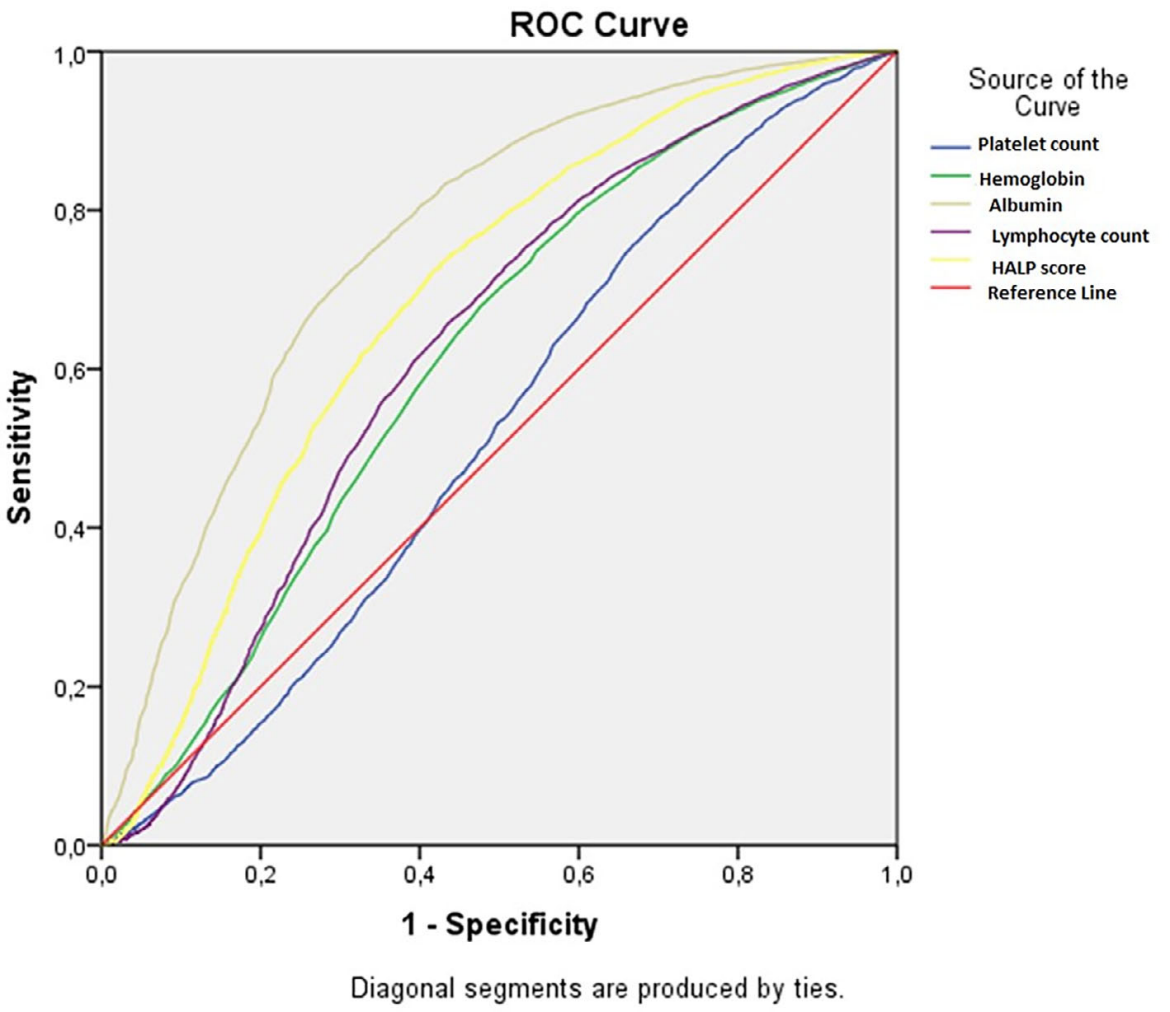
Receiver operating characteristic (ROC) analysis for predicting patient outcomes. HALP, Hemoglobin, Albumin, Lymphocyte and Platelet.

On analyzing the utility of platelet count, lymphocyte count, albumin and hemoglobin levels measured at the time of emergency department presentation for predicting patient outcomes, it was observed that the albumin level at presentation was the most effective predictor of patient outcomes. After albumin, the HALP score, which involved the evaluation of all parameters simultaneously, emerged as the second most valuable predictor of patient outcomes based on receiver operating characteristic analysis (Fig. 2, Table 3).

**Table 3:** Optimal cut‐off points of the Hemoglobin, Albumin, Lymphocyte and Platelet score for predicting patient outcomes

Parameter	Cut off	AUC	*P*‐value	Sensitivity (%)	Specificity (%)	95% CI
Platelet count	219.5	0.520	<0.001	59.5	45.1	0.509–0.532
Hemoglobin	12.5	0.615	<0.001	70.8	49.3	0.603–0.626
Albumin	3.7	0.762	<0.001	60.3	77.6	0.752–0.771
Lymphocyte count	1.31	0.624	<0.001	61.0	60.7	0.612–0.635
HALP score	21.2	0.685	<0.001	70.1	60.0	0.674–0.696

AUC, area under the curve; CI, confidence interval; HALP, Hemoglobin, Albumin, Lymphocyte and Platelet.

## Discussion

To the best of knowledge, this study represents the first investigation in the literature evaluating the ability of the HALP score to predict mortality among geriatric patients. We determined that the HALP score calculated at the time of the emergency department presentation was valuable in predicting mortality in this population.

There is a lack of extensive research on the relationship between the cut‐off value of the HALP score in healthy individuals. However, in a cross‐sectional study involving 8245 healthy volunteers, Antar *et al*. reported an average HALP score of 49 for adults, which was inversely related to the number of chronic diseases present in the volunteers.^9^ The authors also noted that advancing age was associated with a decrease in the HALP score, reporting an average HALP score of 45.6 for healthy volunteers aged ≥65 years.^9^

Although all patients included in the present study were aged ≥65 years, our evaluation did not include an assessment of comorbidities among the enrolled patients. In our study, the average HALP score for the 62 262 included patients aged ≥65 years was 37. The lower HALP scores observed in our study might be associated with undiagnosed chronic conditions among our patients. Furthermore, Antar *et al*. focused on healthy adults, whereas the present study group consisted of patients presenting to the emergency department. The HALP score was notably lower among patients who died in the hospital compared with those who were discharged, which is attributable to the lower albumin and hemoglobin levels of this population at the time of emergency department presentation. The association between the HALP score and mortality stems from the parameters that constitute this score. The first of these parameters is hemogram. In the geriatric patient group, anemia has been associated with a range of issues, including cognitive decline and dementia, frailty, increased risk of falls, decreased functional capacity, depression, prolonged hospital stays, and early death.^10^ Therefore, low hemoglobin levels could contribute to mortality by causing tissue‐wide hypoxia.

The second parameter that constitutes the HALP score is albumin, which is associated with nutritional status and is noteworthy for its negative acute phase reactant properties in inflammatory conditions.^11^ In response to the increased synthesis of positive acute phase proteins, the synthesis of negative acute phase proteins, such as albumin and transferrin, is suppressed.^12^ Therefore, low albumin levels are important in evaluating geriatric patients due to the presence of both malnutrition and increased inflammatory markers. In the present study, we evaluated geriatric patients who presented to the emergency department with active diseases; therefore, we observed lower albumin levels in those who had a fatal outcome associated with inflammatory conditions. Systemic inflammation is assumed to stimulate neutrophilia and lymphopenia.^13^ Ayrancı *et al*., who evaluated geriatric patients presenting to the emergency department with active diseases, also found a low lymphocyte ratio in the group with mortality compared with the group without mortality.^14^ In the present study, consistent with the literature, the lymphocyte count was lower in patients who developed mortality.

Another parameter constituting the HALP score is platelet count. Platelets are known for their hemostatic functions, as well as their role in immune response and inflammatory processes. They engage in complex interactions with various immune‐inflammatory cells, facilitating the aggregation of lymphocytes at the site of inflammation in damaged vascular areas, actively mediating host responses during bacterial infections and interacting with neutrophils during viral infections, thereby influencing responses that vary according to the nature and duration of the infection.^15^ Maintenance of these complex and diverse activation states is essential for vascular homeostasis and health regulation.^15^ Consequently, elevated platelet levels or markers of increased platelet activation have been associated with mortality in critical diseases.^16^, ^17^ However, some studies have found lower platelet levels to be more valuable in predicting mortality in critically ill patients.^18^, ^19^ In the present study, platelet levels were similarly found to be lower in patients with mortality. However, possibly due to the lymphocyte level decreasing more significantly than the platelet level, the reduction in the platelet level did not result in a substantial increase in the HALP score.

A strong aspect of the present study is its large number of patients. However, it also had some limitations. First, there was variability between the initial diagnosis at the time of emergency department presentation and the diagnoses made during follow up. Second, HALP scores were not calculated separately for different diagnoses. Consequently, the primary diagnoses of the patients might have affected the HALP score. Third, the chronic comorbidities of the patients included in the study were not evaluated. Finally, the study was carried out in a single center with a retrospective design.

The individual assessment of hemoglobin, albumin, lymphocyte and platelet levels can predict mortality in geriatric patients presenting to the emergency department. However, the HALP score, which incorporates all these parameters, appears to be a valuable tool in predicting mortality in this patient population. Geriatric patients initially presenting with a low HALP score should be considered at increased risk for mortality, and might benefit from hospitalization and further evaluation by a geriatric specialist rather than being discharged from the emergency department.

## Funding information

No financial support was received from any institution or organization for this study.

## Disclosure statement

The authors declare no conflict of interest.

## Author contributions

1. Study Design: FT. 2. Data Collection: FT, AG, ET. 3. Statistical Analysis: ET, AG, FT. 4. Data Interpretation: AG, FT, ET. 5. Manuscript Preparation: FT, ET, AG. 6. Literature Search: FT, AG, ET. 7. Funds Collection: FT.

## Ethics statement

This study was approved by the Atatürk University Clinical Research Ethics Committee with decision number 4/34 and dated 6 July 2024.

## Data Availability

The data that support the findings of this study are available on request from the corresponding author. The data are not publicly available due to privacy or ethical restrictions.
